# VAR2CSA binding phenotype has ancient origin and arose before *Plasmodium falciparum* crossed to humans: implications in placental malaria vaccine design

**DOI:** 10.1038/s41598-019-53334-8

**Published:** 2019-11-18

**Authors:** Stéphane Gangnard, Arnaud Chêne, Sébastien Dechavanne, Anand Srivastava, Marion Avril, Joseph D. Smith, Benoît Gamain

**Affiliations:** 1 Université de Paris, UMR_S1134, BIGR, INSERM, F-75015 Paris, France; 2 Institut National de la Transfusion Sanguine, F-75015 Paris, France; 3 Laboratory of excellence GR-Ex, F-75015 Paris, France; 4 Seattle Children’s Research Institute, Seattle, WA 98109 USA; 5 Department of Global Health, University of Washington, Seattle, WA 98195 USA

**Keywords:** Parasite evolution, Parasite immune evasion

## Abstract

VAR2CSA is a leading candidate for developing a placental malaria (PM) vaccine that would protect pregnant women living in malaria endemic areas against placental infections and improve birth outcomes. Two VAR2CSA-based PM vaccines are currently under clinical trials, but it is still unclear if the use of a single VAR2CSA variant will be sufficient to induce a broad enough humoral response in humans to cross-react with genetically diverse parasite populations. Additional immuno-focusing vaccine strategies may therefore be required to identify functionally conserved antibody epitopes in VAR2CSA. We explored the possibility that conserved epitopes could exist between VAR2CSA from the chimpanzee parasite *Plasmodium reichenowi* and *Plasmodium falciparum* sequences. Making use of VAR2CSA recombinant proteins originating from both species, we showed that VAR2CSA from *P. reichenowi* (Pr-VAR2CSA) binds to the placental receptor CSA with high specificity and affinity. Antibodies raised against Pr-VAR2CSA were able to recognize native VAR2CSA from different *P. falciparum* genotypes and to inhibit the interaction between CSA and *P. falciparum*-infected erythrocytes expressing different VAR2CSA variants. Our work revealed the existence of cross-species inhibitory epitopes in VAR2CSA and calls for pre-clinical studies assessing the efficacy of novel VAR2CSA-based cross-species boosting regimens.

## Introduction

Placental malaria (PM) is a serious complication of malaria infection associated with accumulation of *Plasmodium falciparum* infected erythrocytes (IEs) in the placental intervillous space^1–3^, leading to adverse health consequences for both mother and child^4^. Pregnant women living in malaria endemic areas gradually acquire antibodies that limit placental infections^5^, thus diminishing the severe clinical outcomes associated with PM. In contrast to parasite populations sequestering in the peripheral microvasculature, which bind to endothelial cell receptors such as CD36, intercellular adhesion molecule 1 (ICAM-1) and endothelial protein C receptor (EPCR)^6–8^, placental IEs bind to an unusually low-sulphated form of chondroitin sulphate A (CSA) found in the placental intervillous spaces^9,10^. The placental binding tropism is mediated by a single *P. falciparum* variant antigen, VAR2CSA, which is expressed at the surface of placental IEs^11–14^. VAR2CSA is an unusually strain-transcendent member of the highly polymorphic *P. falciparum* Erythrocyte Membrane Protein 1 (PfEMP1) family, present in one or more gene copies in every *P. falciparum* genotype^15^. VAR2CSA is a large multidomain protein consisting of six Duffy-binding like (DBL) domains (three DBLx, followed by three DBLε). It also contains a CIDR_PAM_ domain between the DBL2x and DBL3x domains, in a region that is also referred to as interdomain 2 (ID2). The core CSA-binding site in VAR2CSA has been mapped to the DBL2x domain and the flanking interdomain 1 (ID1) and ID2 regions^16,17^.

The N-terminal region of VAR2CSA stands today as a leading candidate for developing a placental malaria vaccine. Recombinant proteins based on the core CSA-binding site in VAR2CSA can elicit adhesion-blocking antibodies and pre-clinical assessments of the two most advanced VAR2CSA-based vaccine candidates PAMVAC and PRIMVAC (ClinicalTrials.gov identifiers NCT02647489 and NCT02658253, respectively)^18^ have paved the way for the clinical development of a vaccine that could protect women against PM^19–21^. However, it is unlikely that a single *P. falciparum*-derived VAR2CSA variant will be sufficient to induce a broad enough humoral response in humans to cross-react with the genetically diverse parasite populations present in endemic areas. Like other highly polymorphic vaccine targets, such as influenza and HIV, immuno-focusing vaccine strategies may be required to target antibodies on functionally conserved epitopes in VAR2CSA.

One of the closest *P. falciparum* relative is the chimpanzee parasite *Plasmodium reichenowi*^22^. Both parasite species belong to the *Laverania* sub-genus and share extensive gene organization and orthology, including the presence of *var*-like genes in *P. reichenowi*^23,24^. Although little is known about the encoded cytoadhesive properties of the *var*-like genes in most *Laverania* parasites, analysis of CIDRα0 and CIDRα1 recombinant domains from *P. reichenowi* has provided evidence that the CD36 and EPCR adhesion traits arose in an ancestral species^24^ prior to chimpanzee and human speciation. Moreover, genome sequencing of all known *Laverania* members revealed that var2csa is most likely a vestige of the ancestral gene family that has been maintained in *P. falciparum* and *P. reichenowi*^25^. Notably, the *var2csa*-like gene in the genome reference *P. reichenowi* CDC strain is annotated as a pseudogene and encodes a truncated protein (NTS-DBL1x-ID1-DBL2x-truncated ID2). In addition to its role in placental cytoadhesion, the *P. falciparum var2csa* gene has been proposed to be a central intermediate in *var* gene switching during antigenic variation^26^. The biological role(s) played by VAR2CSA in ape parasites and the potential clinical consequences for the natural host are still unknown. In order to investigate the evolutionary history of CSA-binding determinants, we performed a functional characterization of VAR2CSA from *P. reichenowi* (Pr-VAR2CSA) and also assessed the conservation of VAR2CSA antibody epitopes, which could have been conserved after evolutionary radiation of the *Laverania* sub-genus. We provide evidence that Pr-VAR2CSA binds to CSA with similar affinity and specificity as VAR2CSA from *P. falciparum* (Pf-VAR2CSA), and that it has the capability to elicit cross-inhibitory antibodies against different CSA-binding *P. falciparum* parasite lines.

## Results

### VAR2CSA from *P. reichenowi* binds to the placental receptor CSA with high specificity and affinity

**Figure 1:**
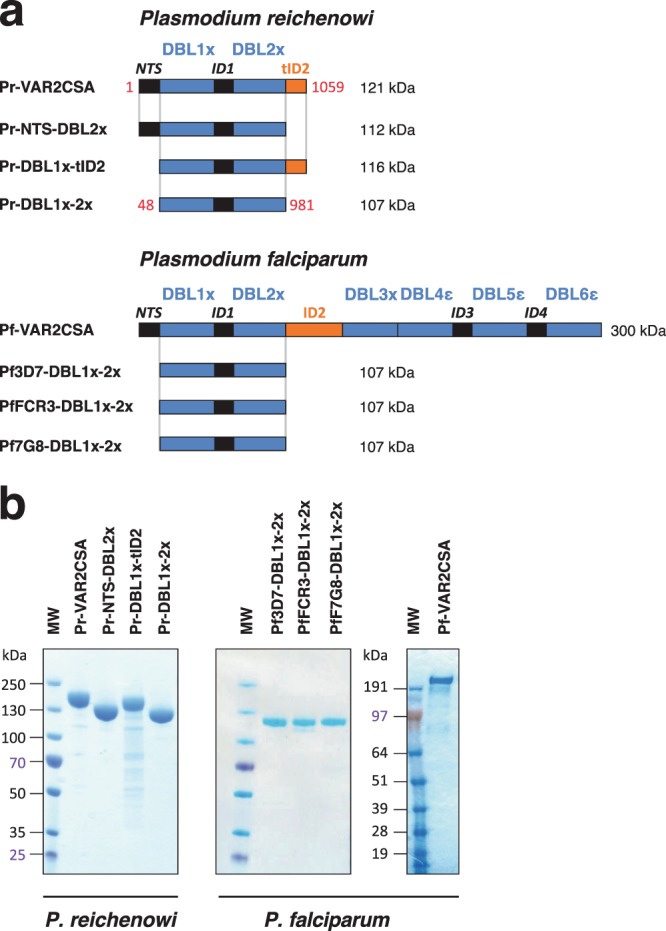
Recombinant proteins used in the study. (**a**) Schematic representation of the VAR2CSA protein organization and sequence boundaries of the multi-domain constructs used in this study. The extracellular part of VAR2CSA from *P. reichenowi* comprises a truncated gene encoding an NTS, two DBL domains (DBL1x to DBL2x) and a truncated ID2 (tID2). The first and last amino-acids of each construct are depicted as they appear within the exon 1 sequence of VAR2CSA from *P. reichenowi*. *P. falciparum* comprises an N-terminal sequence (NTS), six Duffy Binding-Like domains (DBL1x to DBL6ε) interspaced by four inter-domain regions (ID1 to ID4). The ID2 region includes the CIDR_PAM_ domain. **(b)** SDS-PAGE under reducing conditions followed by Coomassie blue staining of the VAR2CSA-derived purified proteins. His-tagged recombinant proteins were purified in a 2-phase process comprising metallo-affinity and gel filtration chromatography. MW: molecular weight. Three independent gels were run and are displayed.

The genome reference *P. reichenowi* CDC strain encodes a truncated VAR2CSA gene that has been annotated as a pseudogene. As compared to *P. falciparum*, the extracellular part of the protein is limited to the NTS-DBL1x-ID1-DBL2x-truncated ID2 (tID2) region (Fig. 1a). Since the CSA-binding region of *P. falciparum* VAR2CSA resides within the ID1-DBL2x-ID2a region of the protein^27^, we hypothesized that *P. reichenowi* VAR2CSA could also harbour functional determinants allowing interaction with glycosaminoglycans. In order to assess if *P. reichenowi* VAR2CSA presents a similar CSA-binding phenotype to that of VAR2CSA from *P. falciparum*, different Pr-VAR2CSA constructs were generated (Fig. 1a**)** and produced as soluble recombinant proteins in HEK cells (Fig. 1b). In addition to a recombinant protein comprising the whole extracellular part of Pr-VAR2CSA, three other protein constructs deprived of either the N-terminal sequence (Pr-DBL1x-ID2) or the tID2 region (Pr-NTS-DBL2x) or lacking both regions (Pr-DBL1x-2x) were designed. The full-length extracellular region of *P. falciparum* VAR2CSA (Pf-VAR2CSA) was also produced as well as three multi-domain constructs comprising the DBL1x-2x part of VAR2CSA and originating from different *P. falciparum* parasite strains (Pf-3D7-DBL1x-2x, Pf-FCR3-DBL1x-2x, Pf-7G8-DBL1x-2x) (Fig. 1a). The Pf-3D7-DBL1x-2x sequence boundaries (aa48-aa981) matches the exact same sequence delimitations of PRIMVAC (Pf-3D7-DBL1x-2x), a leading Pf-VAR2CSA-based PM vaccine currently evaluated in a phase Ia/Ib clinical trial^19,21^. A multiple sequence alignment performed with Pr-VAR2CSA and Pf-VAR2CSA (DBL1x-DBL2x) sequences from the 3D7, FCR3 and 7G8 strains revealed that the *P. reichenowi* protein presents 75.0% identity with Pf-3D7-VAR2CSA, 76.9% with Pf-FCR3-VAR2CSA and 75.4% with Pf-7G8-VAR (Supplementary Fig. S1).

Following a 2-step purification process comprising metallo-affinity and gel filtration chromatography, all recombinant proteins were successfully produced with a high degree of purity as reflected by sodium dodecyl sulphate-polyacrylamide gel electrophoresis (SDS-PAGE) (Fig. 1b). Even though protein integrity was excellent for most of the constructs, slight degradation bands were visible for Pr-DBL1x-tID2 following gel staining with Coomassie blue (Fig. 1b).

**Figure 2:**
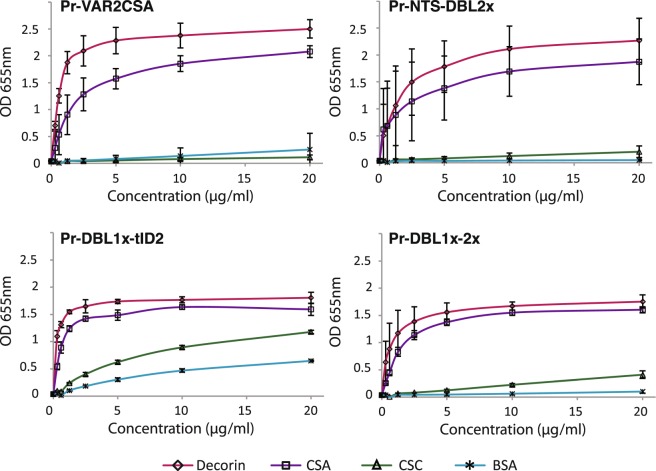
VAR2CSA from *P. reichenowi* binds to the placental receptor CSA with high specificity. ELISA-based direct binding assay of the VAR2CSA-derived constructs to different sulphated glycosaminoglycans. Increasing concentrations of recombinant **(a)** Pr-VAR2CSA, **(b)** Pr-NTS-DBL2x, **(c)** Pr-DBL1x-tID2, **(d)** Pr-DBL1x-2x at serial dilutions of 0.31–20 μg/mL were added to wells previously coated with bovine serum albumin (BSA) or with different glycosaminoglycans; CSA-bearing bovine glycoprotein decorin, chondroitin sulphate A (CSA), chondroitin sulphate C (CSC). Error bars represent the standard deviation of 3 independent experiments.

**Figure 3:**
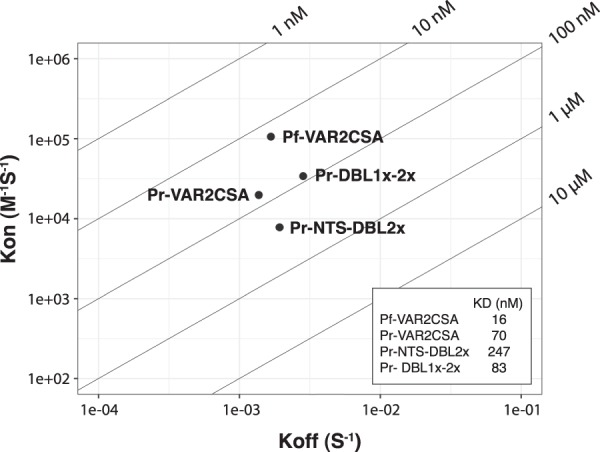
VAR2CSA from *P. reichenowi* binds to the placental receptor CSA with high affinity. RaPID plot of kinetics constants determined by surface plasmon resonance. Koff and Kon values for each analyte are indicated as dots in a 2-dimentional plot. Calculated K_D_ values (Koff/Kon) are depicted in the inset table.

The glycosaminoglycan-binding properties of the different recombinant proteins derived from *P. reichenowi* VAR2CSA were then analysed by direct enzyme-linked immunosorbent assay (ELISA) in which chondroitin sulphate C (CSC), CSA and the CSA-bearing bovine glycoprotein decorin were immobilized on plastic. Bovine serum albumin (BSA) was also used as a non-glycosaminoglycan control protein. PrVAR2CSA, Pr-NTS-DBL2x and Pr-DBL1x-2x specifically bound to CSA and very poorly to CSC or BSA (Fig. 2). In contrast, Pr-DBL1x-tID2 displayed a non-negligible binding to CSC and BSA demonstrating a lower CSA-specificity, as compared to the other constructs. Recombinant protein binding to decorin was consistently higher than binding to purified CSA. Decorin was used in surface plasmon resonance (SPR) experiments to measure the association strength of the different constructs to CSA (Figs 3 and S2). For these experiments, BSA was coated on the reference channel (Fc1) and decorin was immobilized on the analytic channel (Fc2). The specific binding response of the analytes towards decorin was regarded as the Fc2-Fc1 signal. The fitted kinetic data (Koff and Kon) derived from the sensorgrams (Supplementary Fig. S2) are displayed as a RaPID plot in Fig. 3. The affinity constant K_D_ was regarded as Koff/Kon. Pr-VAR2CSA and Pr-DBL1x-2x demonstrated a nanomolar affinity to CSA (K_D_ = 70 nM and 82 nM, respectively), approaching the low nanomolar binding affinity of *P. falciparum* VAR2CSA for the sulphated sugar (K_D_ = 16 nM). In contrast, Pr-NTS-DBL2x had a much weaker interaction with CSA, as compared to the other construct (K_D_ = 247 nM). Taken together, these results provide evidence that VAR2CSA from the chimpanzee malaria parasite *P. reichenowi* possesses functional determinants for specific CSA-binding.

### Anti-Pr-VAR2CSA antibodies recognize VAR2CSA from different *P. falciparum* strains

The existence of CSA-binding determinants in *P. reichenowi* VAR2CSA raises the possibility that functionally conserved antigenic epitopes could exist between VAR2CSA from the chimpanzee parasite *P. reichenowi* and *P. falciparum* sequences. To investigate if cross-species epitopes are present, white rabbits and Wistar rats were immunized with recombinant Pr-VAR2CSA (NTS-DBL1x-ID1-DBL2x-tID2). IgG titrations performed 7 days after the last immunisation showed that Pr-VAR2CSA was more immunogenic in rats than in rabbits as reflected by higher anti-Pr-VAR2CSA IgG antibody titres in the rodent group (Table 1). Rat and rabbit anti-Pr-VAR2CSA antibodies cross-reacted on VAR2CSA-derived recombinant proteins (DBL1x-2x) originating from different *P. falciparum* sequences (3D7, FCR3 and 7G8 strains), thus implying the presence of cross-species epitopes in VAR2CSA. The levels of VAR2CSA-specific antibody obtained towards the *P. falciparum* DBL1x-2x constructs were much lower than Pr-VAR2CSA (10 to 100-fold) suggesting that cross-species epitopes are limited between the *P. reichenowi* and *P. falciparum* VAR2CSA sequences.

**Table 1:** IgG titers (1/) at Day 63.

	Pr-VAR2CSA	Pf3D7-DBL1x-2x	PfFCR3-DBL1x-2x	Pf7G8-DBL1x-2x
Rat 1	5297	323	298	515
Rat 2	43067	489	972	1925
Rat 3	3971	87	54	71
*Mean (SD)*	*17445 (22199)*	*300 (202)*	*441 (475)*	*837 (968)*
Rabbit 1	1879	403	251	281
Rabbit 2	450	55	39	172
*Mean (SD)*	*1165 (1010)*	*229 (246)*	*145 (150)*	*227 (77)*

VAR2CSA immune recognition was then assessed in the context of the native VAR2CSA protein expressed at the surface of *P. falciparum* infected erythrocytes (IEs). Erythrocytes were infected with 3 different parasite lines selected for the CSA-binding phenotype (NF54-CSA, FCR3-CSA and 7G8-CSA) or for the CD36-binding phenotype (FCR3-CD36) as a negative control. The sequence of VAR2CSA from the NF54 strain is identical to the NF54-derived clone 3D7.

**Figure 4:**
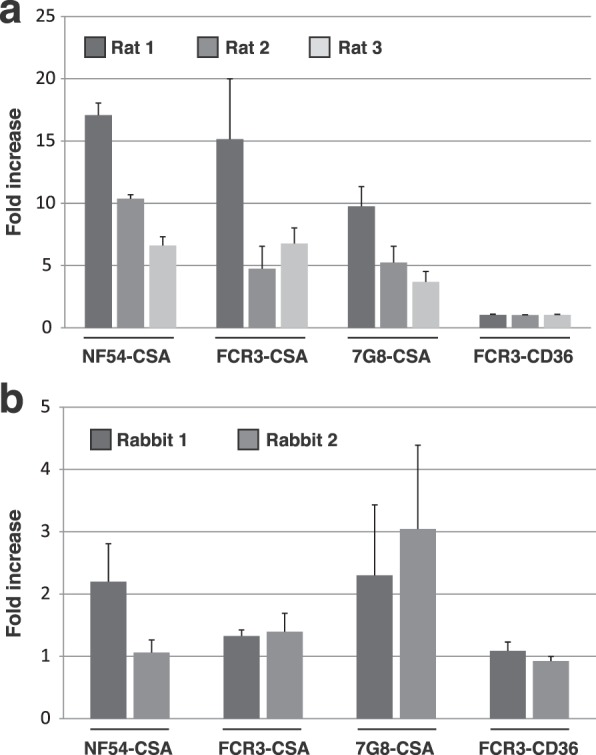
Anti-Pr-VAR2CSA antibodies recognize VAR2CSA from different *P. falciparum* strains. Immune recognition of erythrocytes infected by different *P. falciparum* parasite strains (NF54, FCR3, and 7G8) selected for different adhesive phenotypes (CSA and CD36) by immunisation-induced antibodies directed towards *P. reichenowi* VAR2CSA. Infected erythrocytes were incubated with rat sera **(a)** or rabbit sera **(b)** diluted 1:10. Erythrocyte-bound IgGs were detected using an anti-rat IgG or an anti-rabbit IgG specific antibody PE-conjugated. Cells were then subjected to flow cytometry analysis. Results are expressed as the fold-increase in geometrical mean fluorescence intensity obtained with immune (Day 63) sera compared to pre-immune sera. Error bars represent the standard deviation of 3 independent experiments.

Flow cytometry analysis showed that IgGs present in rat plasma samples were able to recognize IEs expressing different VAR2CSA variants from *P. falciparum* parasites (Fig. 4a). The fold increase in fluorescence signal between immune and pre-immune sera was greater for NF54-CSA (mean fold increase: 11.4) than for FCR3-CSA (8.9) or 7G8-CSA (6.2). The capability of individual rat sera to recognize VAR2CSA varied between animals and did not completely correlate with the antibody titres obtained using the matching DBL1x-2x recombinant proteins. The FCR3-CD36 IE population expressing a non-VAR2CSA PfEMP1 was not recognised by any of the rat samples (Fig. 4a). The rabbit anti-sera were also able to recognize native *P. falciparum* VAR2CSA variants and not the negative control FCR3-CD36 IEs, although at a lower level than the rat sera (two rabbits: mean fold increase NF54-CSA: 1.6; FCR3-CSA: 1.4; 7G8-CSA: 2.7) (Fig. 4b). These results demonstrate the presence of cross-species epitopes within VAR2CSA.

### Anti-Pr-VAR2CSA antibodies inhibit the interaction between CSA and erythrocytes infected with different *P. falciparum* strains

**Figure 5:**
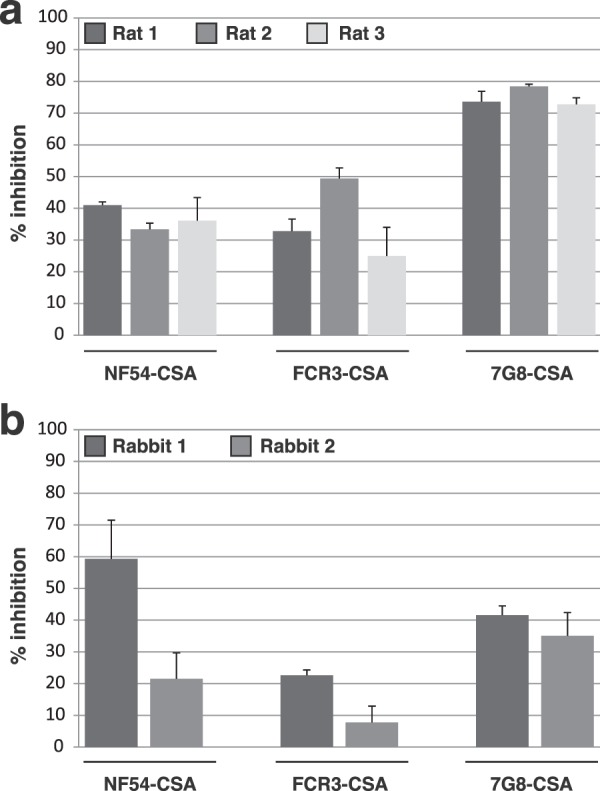
Anti-Pr-VAR2CSA antibodies inhibit the interaction between CSA and erythrocytes infected with different *P. falciparum* VAR2CSA strains. Inhibition of *P. falciparum*-infected erythrocytes binding to CSA by immunisation-induced antibodies directed towards Pr-VAR2CSA. Erythrocytes infected by different *P. falciparum* parasite strains (NF54, FCR3, and 7G8) selected for CSA adhesive phenotype were incubated with rat sera **(a)** or rabbit sera **(b)** diluted 1:10 and then plated on CSA-coated plates. CSA-binding inhibition was assessed by relative quantification of IEs remaining bound to the plate surface. Error bars represent the standard deviation of values obtained from three independent replicates.

The capacity of anti-Pr-VAR2CSA to block the interaction between CSA and *P. falciparum*-infected erythrocytes was assessed in CSA-binding inhibition assays (Fig. 5). The mean percentage of inhibition with rat anti-Pr-VAR2CSA sera was 37% for NF54-CSA, 36% for FCR3-CSA and 75% for 7G8-CSA (Fig. 5a). The mean percentage of inhibition for the rabbit anti-sera was 40% for NF54-CSA, 15% for FCR3-CSA and 38% for 7G8-CSA (Fig. 5b). Taken together this demonstrates that Pr-VAR2CSA was capable of generating cross-species inhibitory antibodies.

## Discussion

Cytoadhesion of *P. falciparum* infected erythrocytes to endothelial cells and the placenta is a major virulence determinant. The main cytoadhesion ligand in *P. falciparum* is the *var* gene family^28^. Increasing lines of evidence from genomic sequencing reveals that the *var* gene family arose in the *Laveranian* sub-genus that infects chimpanzees, gorillas, and humans. Within the *Laveranian* subgenus, clade A and clade B parasites have become specialized for different hosts^23,25,29–31^. While all *Laverania* species retain a two-exon *var* gene structure and are organized into subtelomeric or internal *var* genes, the adhesion domains have diversified between clade A and clade B parasites^25^. In particular, the *var* gene families of the human malaria parasite *P. falciparum* and the chimpanzee malaria parasite *P. reichenowi* highly resemble each other with similar DBL and CIDR sequence types, while different domain types are found in clade A *Laverania* parasites^23,29–31^. In contrast, v*ar* genes are completely absent in non-*Laveranian* plasmodia genomes. Taken together, genomic comparisons strongly suggest that the erythrocytic stage-related cytoadhesion traits originated as a unique adaptation in the *Laverania* sub-genus and then underwent adaptative radiation in different parasite-host combinations. Compared to *P. falciparum*, relatively little is known about the function and biological role of *var* genes in non-human primate hosts.

In *P. falciparum*, *var* genes have diverged to encode proteins that bind the endothelial receptors CD36 and EPCR^8,32–34^. Molecular analysis performed with recombinant CIDR domains from *P. reichenowi* revealed a similar interaction with the human receptors^24^, providing evidence that the CD36 and EPCR binding phenotypes evolved in an ancestral clade B parasite, and has been maintained as a key functional molecular interface in great apes and human hosts. Little is known about the evolutionary origins of other *P. falciparum* cytoadhesion traits.

Notably, nearly all *Laverania* species contain a *var2csa-like* gene^25^, suggesting an extremely ancient origin of this gene and it has been proposed to be a remnant of an ancient multigene family^25^. In *P. falciparum*, VAR2CSA is a placental adhesion ligand^12^ that is present in one or more gene copies in all parasite genotypes^35,36^, and the gene has also been proposed to function as a central intermediate when *var* genes switch during antigenic variation^26^. The core CSA binding region in *P. falciparum* VAR2CSA has been mapped to a region including both the DBLpam2 domain and the flanking ID1 and ID2/CIDRpam regions^16,17^. Although most *Laverania* parasites encode DBL and CIDR domains, the numbers of these domain types differ widely between *Laverania* species^25^. Consequently, the origins of the CSA-binding trait in the *Laverania* parasites and the function of *var2csa*-like gene in primate malaria parasites is unknown.

Here, we showed that *P. reichenowi* VAR2CSA recombinant proteins demonstrated a strong and specific interaction with CSA, comparable to the one of the *P. falciparum* orthologs. We observed similar binding results for different Pr-VAR2CSA constructs except for Pr-DBL1x-tID2 that lacked CSA-binding specificity and presented a weaker binding affinity for the sulphated sugar. However, this recombinant protein contained numerous truncated protein fragments, as compared to the other constructs. Since the Pr-DBL1x-2x recombinant protein presented a similar binding to the full-length Pr-VAR2CSA recombinant protein, it is unlikely that the sole absence of the NTS in Pr-DBL1x-tID2 is responsible for its reduced affinity and specificity. Instead, a structural misfolding due to inappropriate construct boundaries seems more probable to explain the altered Pr-DBL1x-tID2 function. Taken together, our results suggest that the VAR2CSA CSA-binding phenotype has an ancient origin and arose before *Plasmodium falciparum* crossed to humans.

An effective pregnancy malaria vaccine may need to elicit broadly cross-inhibitory antibodies against the VAR2CSA and CSA interaction. However, the extensive VAR2CSA sequence polymorphism poses problems, and there is still limited understanding of whether VAR2CSA contains conserved inhibitory epitopes or how to target them by vaccination. Previous work has shown that the core CSA-binding region in Pf-VAR2CSA (ID1-DBL2-ID2) can elicit adhesion-blocking epitopes, but the DBL2 domain contains several highly polymorphic loops that may contribute to immuno-evasion^37^. For other highly polymorphic vaccine targets, such as HIV and influenza, immuno-focusing vaccine strategies are being evaluated to induce broader neutralizing antibody responses. One strategy is cross-strain boosting with different allelic variants, which has also been evaluated for the CIDR domain in the PfEMP1^38,39^. Here, we demonstrate that Pr-VAR2CSA immunogens are capable of eliciting cross-species inhibitory antibodies. While these epitopes did not dominate the antibody response, they may identify regions that have been functionally conserved between different VAR2CSA alleles. In the future, it will be interesting to characterize the cross-species inhibitory epitopes in Pr-VAR2CSA and to evaluate it in a cross-species boosting regimen to determine if this approach could enhance broadly inhibitory antibody responses.

## Methods

### VAR2CSA-derived proteins expression in HEK 293-F cells

The Pr-VAR2CSA pseudogene sequence encoding residues 1 to 1059 (PlasmoDB PRCDC_0011300) was synthetized with sequence optimization for human codon usage. All N-glycosylation sites were mutated by substitution of the amino-acid asparagine with a glutamine. DNA sequences encoding for Pr-VAR2CSA, Pr-NTS-DBL2x (residues 1–981), Pr-DBL1x-tID2 (residues 48–1059) and Pr-DBL1X‐2x (residues 48–981) were cloned into the expression plasmid pTT3. The Pf-VAR2CSA sequence was identical to the one previously reported^40^ and recombinant protein expression was carried out as previously described^41^. Briefly, FreeStyle 293-F cells (Invitrogen) were grown in Freestyle 293 serum free expression medium and transfected with the pTT3 plasmid containing the synthetic gene. Following a 72 hour-incubation at 37 °C during which the recombinant protein was expressed and released in the medium, the cell culture was centrifuged and the supernatant was harvested for subsequent recombinant protein purification.

### VAR2CSA-derived proteins expression in *Escherichia coli SHuffle*^*®*^

DNA sequences encoding for the Pf-VAR2CSA multi-domains Pf-(3D7)-DBL1x-2x (residues 48–981; PlasmoDB PF3D7_1200600), Pf-(FCR3)-DBL1x-2x (residues 48–981; PlasmoDB PfIT_120006100), Pf-(7G8)-DBL1x-2x (residues 48–981; PlasmoDB Pf7G8_120005700) were amplified from genomic DNA of the *P. falciparum* strains 3D7, FCR3 (IT) and 7G8, respectively and cloned into pET-15b. Recombinant proteins were expressed in SHuffle^®^ bacteria (New England Biolabs) for 20 h at 20 °C following Isopropyl β-d-1-thiogalactopyranoside (IPTG) induction (0.2 mM). All recombinant proteins carried a C-terminal His-tag and were purified in a 2-phase process comprising a metallo-affinity chromatography purification step performed on HisTrap^TM^ columns (GE Healthcare) followed by a size exclusion chromatography step on a Superdex 200 10/300 GL column (GE Healthcare).

### Enzyme-Linked Immunosorbent Assay (ELISA)

ELISA were performed as previously described^16^. Briefly, microtiter plates (Immunolon 4HBX 3855) were coated overnight at 4 °C with different sulfated glycosaminoglycans (GAG): 5 µg/mL for decorin (Sigma, D8428); 50 µg/mL for chondroitin sulfate A (CSA) (Sigma, C8529) and chondroitin sulfate C (CSC) (Sigma, 400670). After blocking, each recombinant protein (Pr-VAR2CSA, Pr-NTS-DBL2x, Pr-DBL1x-tID2 and Pr-DBL1x-2x) in serial dilutions of 0.31–20 µg/mL was added into the wells and incubated for 1 h at 37 °C. His-tagged protein detection was performed using an anti-His HRP-conjugated antibody (Qiagen), diluted 1/2000. Absorbance was measured at 655 nm following addition of TMB (3,3′,5,5′-tetramethylbenzidine) (Biorad).

### Surface plasmon resonance

Interaction between the recombinant proteins and bovine decorin (Sigma, D8428) was studied by surface plasmon resonance (SPR) using a Biacore^®^ X100 system (GE Healthcare). Bovine decorin was biotinylated using EZ-Link™ Sulfo-NHS-LC-Biotin kit (ThermoFischer Scientific) and immobilized on a SA sensor chip (GE Healthcare). The amount of immobilized decorin corresponded to 268 Response Units (RU). A separate flow channel on the same sensor chip, reserved for control runs, was prepared in the same way but with biotinylated BSA, immobilized at a level of 685 RU. Analytes were injected in dilution series at 25 °C and at a flow rate of 20 μL.min^−1^. Between each injection, surfaces were regenerated by 2 washes with 5 μL of 2 M NaCl followed by 1 wash with 1 M NaCl/50 mM NaOH. All curves were corrected for nonspecific binding by subtraction of control curves obtained from injection of the corresponding protein through the blank flow channel. Kinetic constants were determined following a 1:1 binding curve fitting.

### Animal immunization

Small animal immunizations with the Pr-VAR2CSA recombinant protein were performed by Biotem (Grenoble, France) according to animal immunization guidelines. All animal immunization experiments were executed in strict accordance with good animal practices, following the EU animal welfare legislation and after approval of the Biotem (France) ethical committee. Every effort was made to minimize suffering.

Two white rabbits and three Wistar rats received each 4 injections of Pr-VAR2CSA (50 μg in PBS) in combination with Freund adjuvant following the immunization procedures described in Supplementary Fig. 3. Animals were bled 7 days after the last immunisation (day 63). Sera samples were then collected and stored at −20 °C until experimental analysis.

### IgG titers

IgG titers determination was performed as previously described^21^. Briefly, microtiter plates (Immunolon 4HBX 3855) were coated overnight with 1 μg/ml antigen. After removal of blocking solution, rabbit or rat sera serial dilutions were added into the wells and incubated for 1 h at 37 °C. Anti-rabbit or anti-rat IgG (Fc-specific) HRP-conjugated antibody (Jackson Immunoresearch 111-036-046 or 112-036-071, respectively) at 1/2000 were used for detection of bound-IgGs. Absorbance was measured at 655 nm following addition of TMB. After data plotting and 4-parameter logistic regression curve fitting, the plasma dilution corresponding to 50% of the maximal optical density (OD) value (sigmoid curve plateau) was regarded as the antigen specific antibody titer.

### Parasite culture

Parasite culture was carried out as previously described^21^. Erythrocytes infected with NF54, FCR3 (IT4) and 7G8 were selected for the CSA-binding phenotype by multiple panning rounds on CSA (Sigma). These selected populations are referred to as NF54-CSA, FCR3-CSA and 7G8-CSA. Erythrocytes infected with FCR3 were also selected for the CD36-binding phenotype by multiple panning rounds on recombinant CD36 (R&D systems) and are referred to as FCR3-CD36.

### Flow cytometry

VarioMACS (Miltenyi Biotec) purified IEs at mid/late trophozoite stage were resuspended in PBS 0.2% BSA and counted. For each assay, 2.5 × 10^5^ IEs were washed in PBS and incubated with the sera (previously depleted against normal erythrocytes) diluted 1/10 in PBS 0.2% BSA for 1 h at 4 °C. IEs were washed twice with PBS and resuspended in 100 µl of PE conjugated goat anti-rabbit or goat anti-rat IgG diluted 1/100 in PBS 0.2% BSA for 60 min at 4 °C. After washing twice in PBS 0.2% BSA, IEs were resuspended in paraformaldehyde 2% in PBS and kept at 4 °C overnight in darkness. Cells were then washed twice with PBS and analyzed by flow cytometry using a BD FACScanto II flow cytometer with the Flow Jo 10.0 software. Parasite nuclei were stained with Topro3 (1/4,000 dilution) (ThermoFischer Scientific). The results are expressed as the ratio between the geometric mean fluorescence intensities (MFI) of the immune sera and the respective non-immune sera.

### CSA-binding inhibition assays

CSA-binding inhibition were performed as previously described^21^. Briefly, microtiter plates were coated overnight with CSA (Sigma) at 1 mg/ml or BSA (Roche) at 1%. Purified infected erythrocytes (trophozoite stage) were pre-incubated with plasma samples diluted in RPMI 2% FBS (1/10) for 1 h at 37 °C. Infected erythrocytes were then added into the pre-coated wells (10^6^ cells; 100 μl/well) and incubated for 1 h at 37 °C. Plates were then washed three times and infected erythrocytes remaining attached to the surface were lysed by addition of TMB. Absorbance was measured at 655 nm. For rabbits, results were expressed as % of inhibition of the post-immunization samples compared to the respective pre-immunization samples. Due to limited amounts of pre-immune samples, rat results were expressed as % of inhibition of the post-immunization samples compared to medium alone.

## Supplementary information

Supplementary information

## Data Availability

The authors confirm that the data supporting the findings of this study are available within the article and its Supplementary Material.
