# Supplementary material for: VAR2CSA binding phenotype has ancient origin and arose before Plasmodium falciparum crossed to humans: implications in placental malaria vaccine design
Source: Sci Rep. 2019 Nov 18;9:16978. doi: 10.1038/s41598-019-53334-8 (PMC6861233; doi:10.1038/s41598-019-53334-8)

VAR2CSA binding phenotype has ancient origin and arose before *Plasmodium falciparum* crossed to humans: implications in placental malaria vaccine design

Stéphane Gangnard, Arnaud Chêne, Sébastien Dechavanne, Anand Srivastava, Marion Avril, Joseph D. Smith, Benoît Gamain

SUPPLEMENTARY INFORMATION

SUPPLEMENTARY FIGURE S1

a. ClustalW multiple sequence alignment of DBL1x-2x

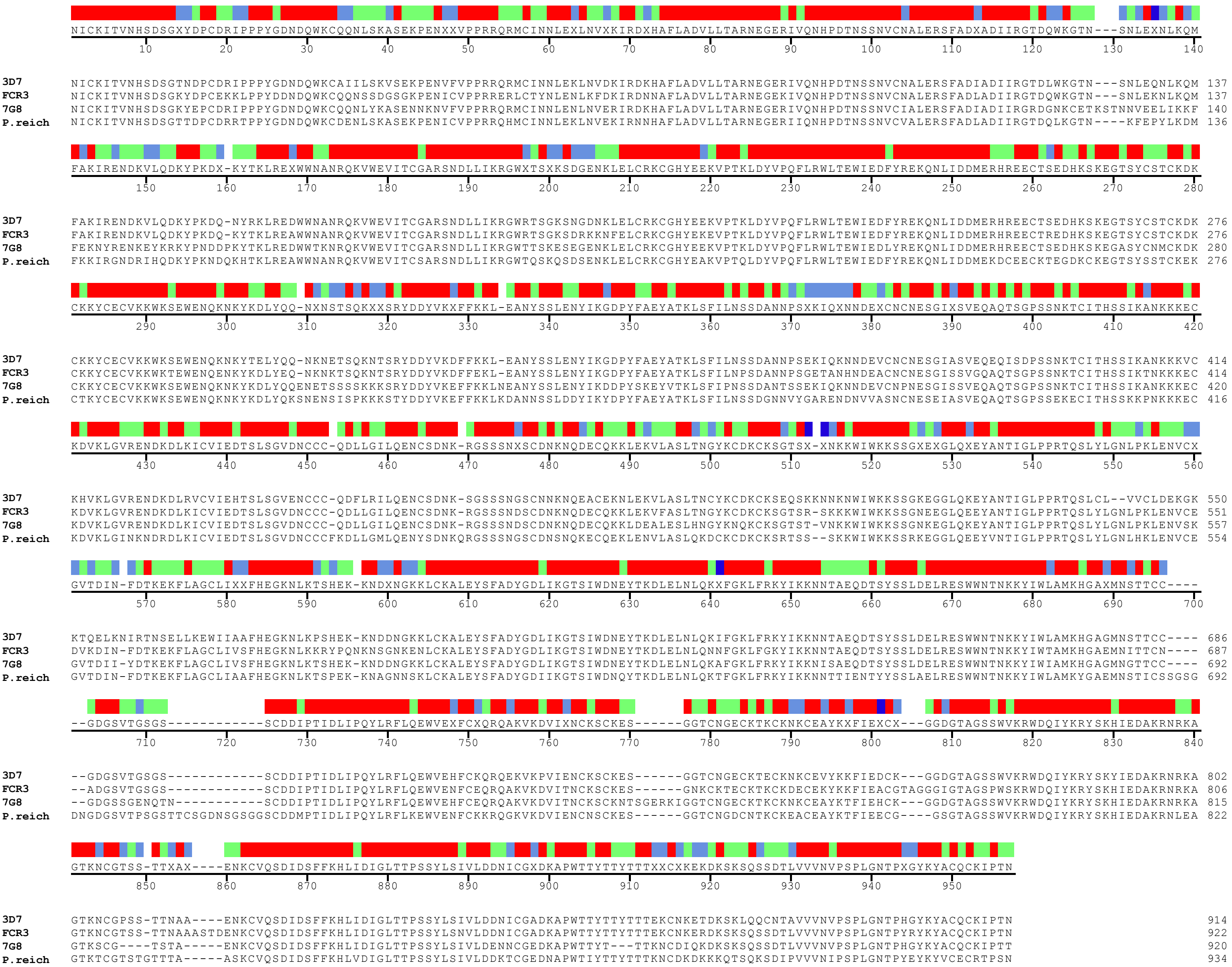

b. Sequence pair distances of DBL1x-DBL2x - percent Identity

	Pf3D7-DBL1x-2x	PfFCR3-DBL1x-2x	Pf7G8-DBL1x-2x
Pr-VAR2CSA	75%	76.9%	75.4%
Pf3D7-DBL1x-2x		82.2%	80.9%
PfFCR3-DBL1x-2x			80.2%

SUPPLEMENTARY FIGURE S2

Surface Plasmon Resonance sensorgrams

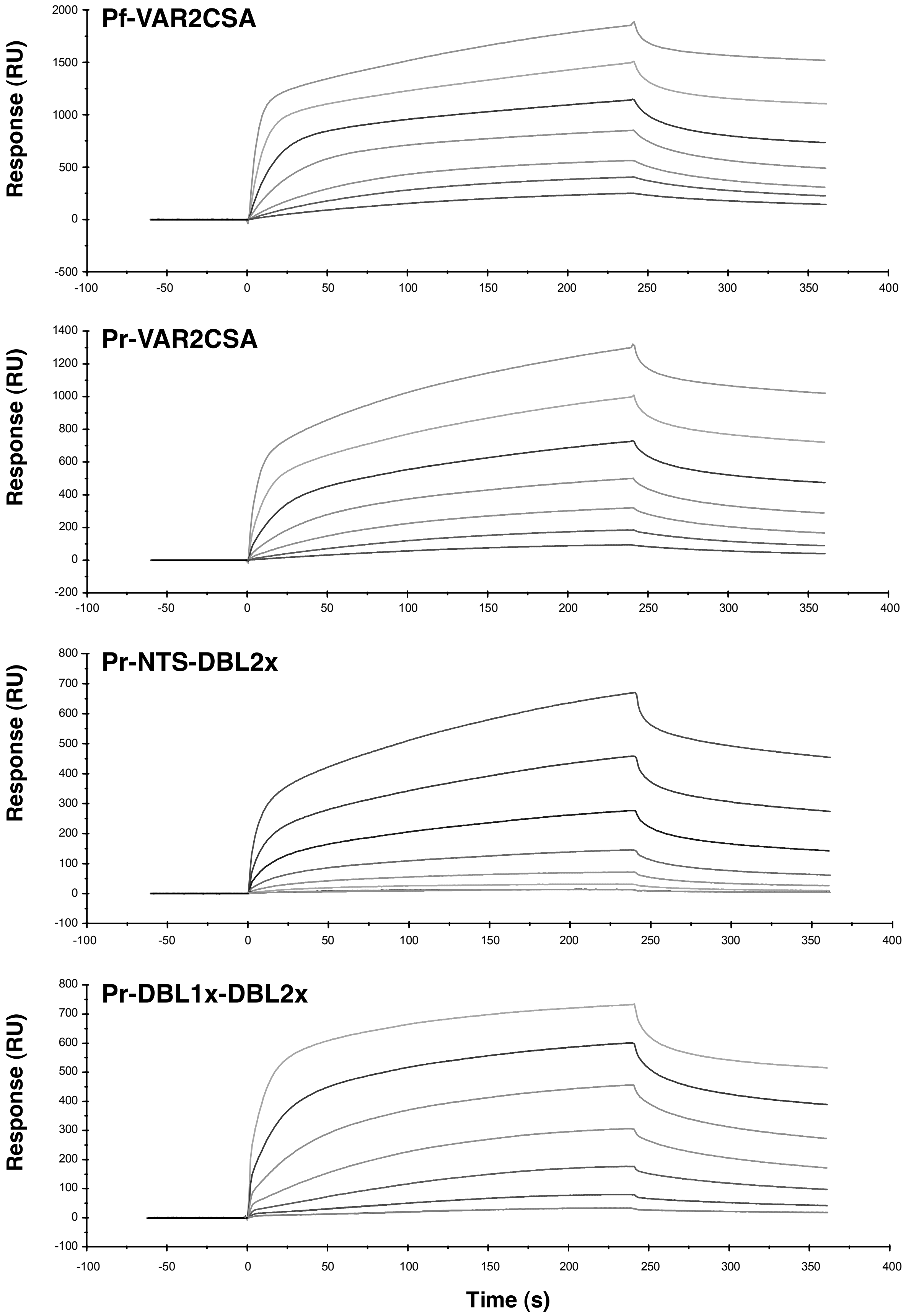

The CSA-carrying protein decorin was immobilized on a SA chip.
Analytes were injected at different concentrations ranging from11 nM to 0.75 M.

SUPPLEMENTARY FIGURE S3

Immunization scheme with Pr-VAR2CSA
Rabbits and Rats

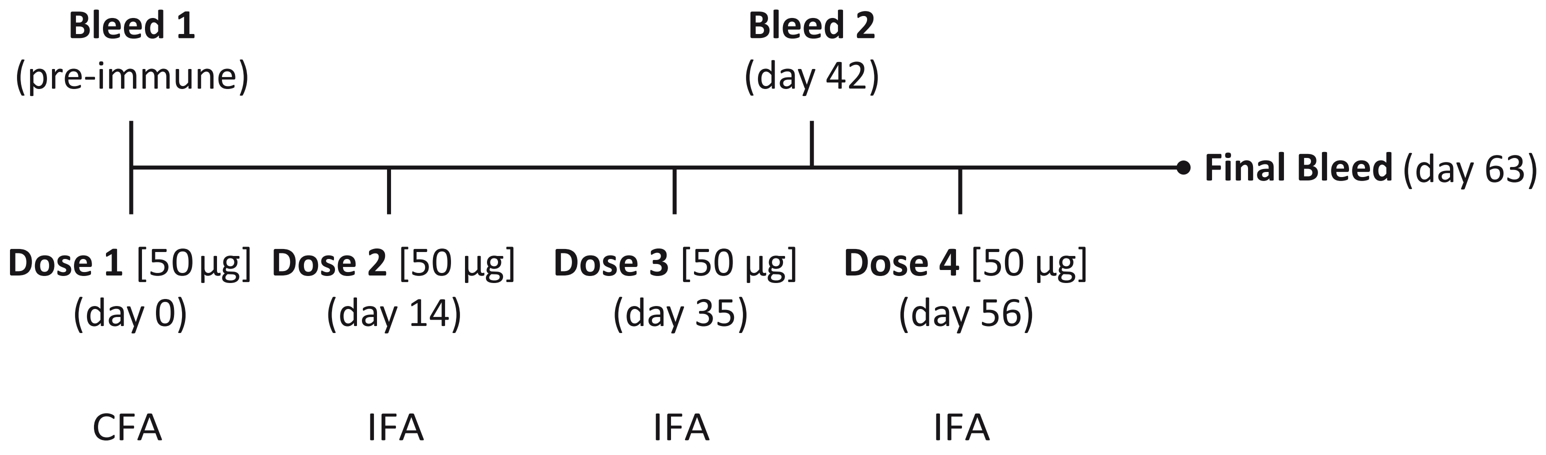

CFA: Complete Freund Adjuvant
IFA: Incomplete Freund Adjuvant

FULL-LENGTH ORIGINAL GELS FROM FIGURE 1B

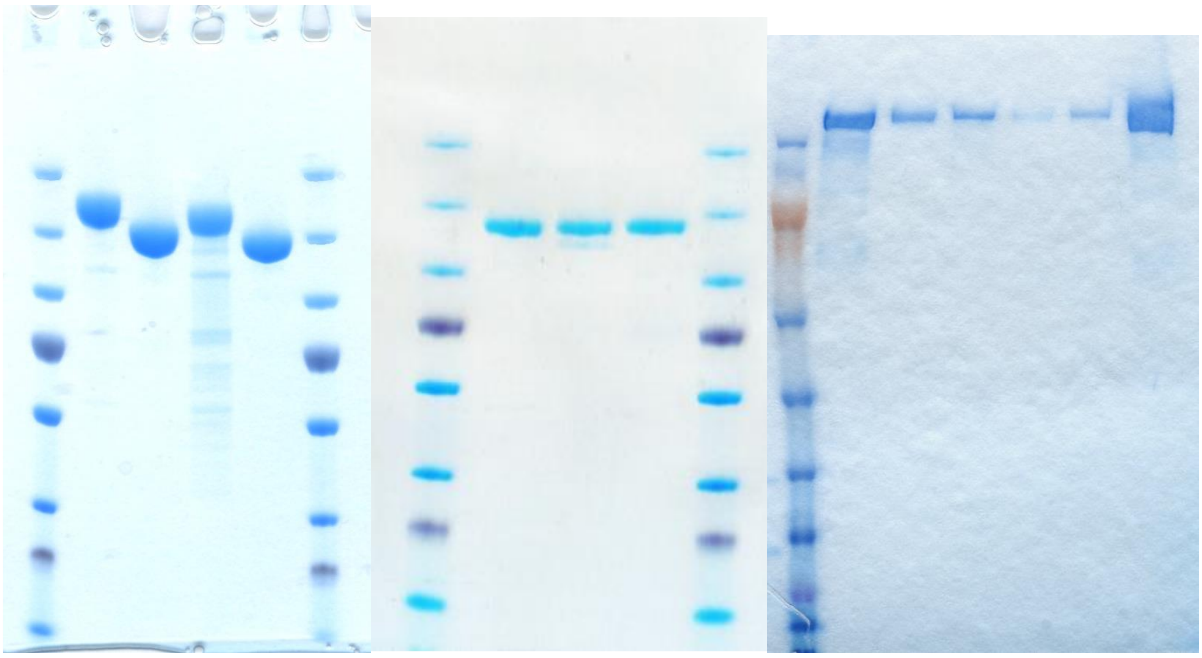

Supplement: Supplementary file 1 — Supplementary information [file 41598_2019_53334_MOESM1_ESM.pdf]
